# A Dominant Social Comparison Heuristic Unites Alternative Mechanisms for the Evolution of Indirect Reciprocity

**DOI:** 10.1038/srep31459

**Published:** 2016-08-12

**Authors:** Roger M. Whitaker, Gualtiero B. Colombo, Stuart M. Allen, Robin I. M. Dunbar

**Affiliations:** 1 Cardiff University, School of Computer Science and Informatics, 5 The Parade, Roath, Cardiff, CF24 3AA, UK; 2 University of Oxford, Department of Experimental Psychology, 9 South Parks Road, Oxford, OX1 3UD, UK

## Abstract

Cooperation is a fundamental human trait but our understanding of how it functions remains incomplete. Indirect reciprocity is a particular case in point, where one-shot donations are made to unrelated beneficiaries without any guarantee of payback. Existing insights are largely from two independent perspectives: i) individual-level cognitive behaviour in decision making, and ii) identification of conditions that favour evolution of cooperation. We identify a fundamental connection between these two areas by examining social comparison as a means through which indirect reciprocity can evolve. Social comparison is well established as an inherent human disposition through which humans navigate the social world by self-referential evaluation of others. Donating to those that are at least as reputable as oneself emerges as a dominant heuristic, which represents aspirational homophily. This heuristic is found to be implicitly present in the current knowledge of conditions that favour indirect reciprocity. The effective social norms for updating reputation are also observed to support this heuristic. We hypothesise that the cognitive challenge associated with social comparison has contributed to cerebral expansion and the disproportionate human brain size, consistent with the social complexity hypothesis. The findings have relevance for the evolution of autonomous systems that are characterised by one-shot interactions.

Insights have long been sought as to how indirect reciprocity has evolved in the human population. Indirect reciprocity can be modelled through prosocial donations which result in a cost *c* to the donor and a benefit *b* for a genetically unrelated recipient, where *b* > *c* > 0. Widely used^1^,^2^,^3^,^4^,^5^,^6^ to consider indirect reciprocity^7^,^8^, this model is a subclass of the mutual aid game^9^ where the donor incurs a cost with no guarantee of reciprocation from the beneficiary, or any other individual. Such prosocial behaviour is widespread in human society^10^, influencing diverse phenomena such as morality^11^, culture^12^, economics and technology^13^.

Precisely why humans donate their resources to unrelated individuals has received considerable attention but remains only partially understood. Hamilton’s kin selection theory^14^,^15^ indicates how it is expected of kin, but this does not extend to unrelated strangers. Further contributions have addressed two perspectives. Firstly, from early contributions on sustaining cooperation^15^,^16^, research has sought to characterise the conditions through which evolution promotes indirect reciprocity^1^,^4^,^9^,^17^,^18^, extending to the coevolution of genes and social norms^19^,^20^,^21^. More recently, the focus has been on the cognitive characteristics of prosocial decision making^22^,^23^, addressing the extent to which prosocial behaviour is intuitive^24^ and mediated as a heuristic within a framework of dual cognitive processing^25^,^26^.

This recent examination of prosocial decision making is built on the dominance of intuitive processes^27^ that allow complexity to be handled with a low cognitive burden. These processes represent heuristics which are fast and automatic, triggered by cues, guided by emotion and association, and involve little conscious thought^28^. Such type-1 cognitive processes are distinguished from deliberate reasoning: in contrast the alternative (type-2) cognitive processes are slower, reflective and present a greater cognitive challenge. The Social Heuristics Hypothesis^22^ proposes that dual-processing governs intuitive prosociality: the behaviours that support success in regular social interactions become intuitive and automatic, unless they are moderated by reflective type-2 processes that represent learning to update a type-1 heuristic.

Based on evidence^22^ that the dual processing framework may shape intuitive prosocial behaviour, an immediate question concerns the nature of possible type-1 heuristics, and their general characteristics. In the absence of detailed context and extensive memory, perception relative to oneself provides an immediate and persistent frame of reference^29^. Stemming from the seminal contributions on social comparison by Festinger^30^, there is comprehensive evidence^31^,^32^,^33^,^34^ that self-referential evaluation influences decision making under conditions of bounded rationality, and from a social perspective, comparison enables generosity to be influenced by the actions of others^35^,^36^,^37^. Consequently, there is a basis to suggest that social comparison is a potential feature in type-1 heuristics, as suggested in other contexts^38^,^39^. Social comparison is also phylogenetically ancient^40^ and embedded in human survival, with its suggested origins in evaluating competitors and assessing whether or not to commit resources to challenge a rival in the hierarchy. Continued re-assessment of others and deliberation over observed social positioning requires significant cognitive resources relative to other species^7^, consistent with the challenge of type-2 processing and the unusually large brain size in humans compared to other all other vertebrates^41^.

But what are the successful social comparison heuristics and how does social comparison feature in prosocial behavioural strategies? Simulation is a well established methodology to assess the evolution of cooperation^16^. In isolation of other factors, evolutionary simulation allows us to examine the social comparison heuristics favoured by natural selection, and the consequences of strategies that incorporate social comparison heuristics. We assess this in the context of indirect reciprocity and the donation game, where reputation acts as a universal currency^42^ through which social credibility between non-kin can be displayed, assessed and acted upon^7^,^8^,^43^,^44^,^45^,^46^. Specifically, we consider the self-comparison of reputation as a basis for heuristic decision making concerning donation.

To model social comparison we may assume that a donor *i* assesses the reputation *r*_*j*_ of a potential recipient *j*, against their own reputation, *r*_*i*_, with three possible outcomes, establishing either: approximate similarity (*r*_*j*_ − Δ ≤ *r*_*i*_ ≤ *r*_*j*_ + Δ), upward self-comparison (*r*_*j*_ > *r*_*i*_ + Δ), or downward self-comparison (*r*_*j*_ < *r*_*i*_ − Δ). Reputation is assumed to be public and available to all agents. After assessing the potential recipient *j*, the donation decision that *i* makes in respect of *j* depends on their choice of social comparison heuristic. The social comparison heuristic for an individual *i* is represented as a triple of binary variables (*s*_*i*_, *u*_*i*_, *d*_*i*_) indicating whether or not *i* donates when similarity (*s*_*i*_), upward comparison (*u*_*i*_) or downward comparison (*d*_*i*_) is observed by *i* in respect of *j*’s reputation. For example, (1, 1, 0) indicates that *i* would donate to *j* exactly when *i* observes either approximate similarity or upward comparison of reputation in respect of *j*. Further, (0, 1, 1) indicates that *i* would donate to *j* precisely when the reputation of *j* is not approximately similar to that of *i*, and so on. Consequently there are 2^3^ possible social comparison heuristics that an individual may adopt.

Despite the potentially significant role that social comparison plays in human behaviour, social comparison has rarely featured in the evolutionary analyses of indirect reciprocity. In evolutionary terms, social comparison heuristics represent action rules. These operate in tandem with assessment rules that are the criteria by which the donor’s reputation is updated in light of their actions, and a combination of action and assessment rules represents a strategy. Assessment rules represent social norms, which humans are well-disposed to internalising and perpetuating^20^,^47^,^48^ with the judgement over reward and penalty that they provide formulating a model for morality^7^. Assessment rules are also highly influential in evolution, with three main alternatives studied being *image scoring*, *standing* and *judging*, with additional variations on these^49^.

Early work exploring indirect reciprocity tended not to have strict delineation between action and assessment rules. *Standing*^9^ was such a breakthrough, which identified the conditions through which indirect reciprocity may evolve from pairwise application of the donor game, showing that “tit-for-tat” behaviour supporting the evolution of direct reciprocity^50^ can be generalised through standing. This assessment rule effectively classifies each individual in the population as either good or bad, penalising the good if they donate to the bad. *Image scoring*^1^,^8^ was the first significant alternative, involving a simple assessment rule where reputation is incremented or decremented in response to donation or defection. A limitation of image scoring is that discriminators who choose not to cooperate with defectors may be unfairly labelled as less cooperative^3^,^17^. Consequently, with their roots in the work of Sugden^9^, *standing*^3^ and *judging*^51^,^52^ have emerged as the natural alternatives that capture “legitimate shirking”^18^,^47^,^53^. These discrimination rules have mainly been studied assuming that reputation has a binary representation^4^,^52^,^54^. However non-binary reputation permits a greater range in status from which the donor can make assessments of others.

To investigate social comparison in the presence of a non-binary reputation, we generalise standing and judging as defined for binary representation. We decrement reputation when defection occurs in light of a request from a player whose reputation is not lesser than that of the donor’s reputation, with the additional requirement for judging that reputation is decremented when cooperation occurs in light of a request from a less reputable player. Otherwise reputation is incremented when the donor cooperates and decremented when the donor defects.

We examine the evolution of social comparison heuristics in the presence of alternative assessment rules, and observe the self-comparison heuristics that are promoted by natural selection. We model a population of *N* agents from which random pairs are selected to play the donation game. Each generation involves playing *m* rounds of the donation game, and in each game a player pair *i*, *j* is randomly selected from the population. Player *i* chooses whether or not to donate to *j* based on its current social comparison heuristic. If *i* chooses to donate then the total payoffs for *i* and *j* are updated, with *i* incurring a cost *c* and *j* gaining a benefit *b*. After each game, the reputation for *i* is updated in light of their donation behaviour, in accordance with either image scoring, standing or judging. After completing *m* rounds of the donation game, the next generation is created through asexual reproduction. Social comparison heuristics are propagated to the next generation of agents based on uniform random selection weighted by their relative payoff, with mutation allowing for a random change of heuristic.

Unless otherwise stated, our results assume a single homogeneous population, however we also investigate the effects of having a structured population, where agents only undertake interactions within sub-groups. Genetic consideration of such a heterogeneous population originates from a spatial perspective through the Island Model^55^. More recently in an online context, such self-focussed sub-groups have been found to cause significant disruptive effects^56^. Where indicated, we apply an idealised Island Model^17^ in which the population is sub-divided into *g* social groups. This model restricts players to in-group interactions and the reproductive influence of the global population is controlled as an experimental parameter. Further details are provided in the methods section.

## Results

### The similar and upward social comparison heuristic dominates

**Figure 1:**
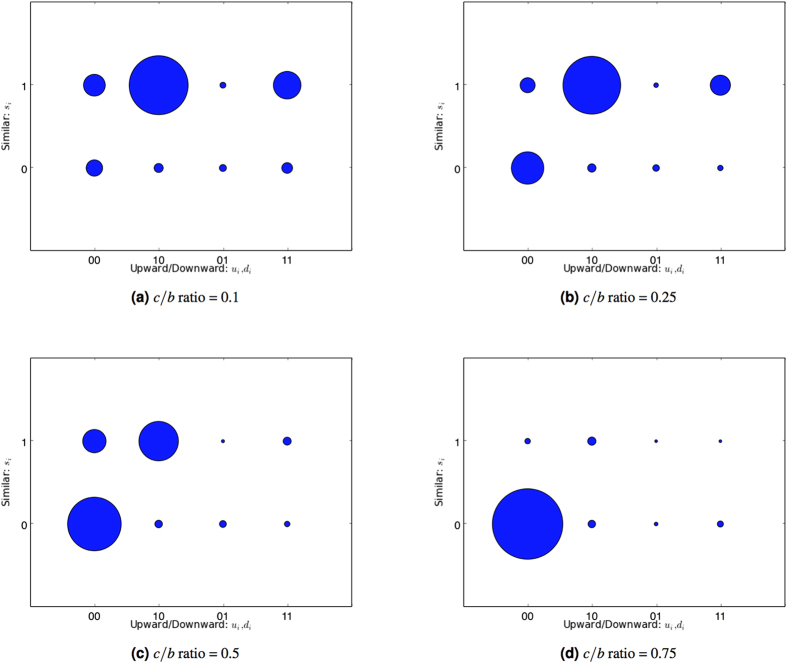
Evolution of social comparison heuristics with image scoring assessment while varying the cost-benefit ratio *c*/*b*. The plots represent the relative distribution of heuristics present in the population taken from all generations. The shaded areas are proportional to the frequency of the associated heuristic. Parameters settings are reported in the Methods Section.

Initially we consider the effect of social comparison using the image scoring assessment rule, which is the least sophisticated approach that allows observation of evolution without any effects from discriminatory assessment. Keeping other variables constant, we vary the cost-benefit ratio *c*/*b* as shown in Fig. 1.

Low *c*/*b* ratios, such as 0.1, are typically required for indirect reciprocity to be sustained through image scoring models^17^, and when the cost-benefit ratio reaches 0.5, they are known to perform quite poorly. Figure 1 reflects this; however in the cost-benefit range where evolution is sustained (i.e., at most *c*/*b* = 0.25), the (1, 1, 0) heuristic, representing *donation to those with similar or better reputation*, clearly dominates. This indicates a potential cycle of donation that is driven by an escalating relative perception.

An individual *i* who frequently donates will experience an increase in their own reputation, which affects their perception of others relative to themselves. For example, after a reputational increase for *i*, a third party *j* who originally had a similar reputation to *i* is subsequently perceived by *i* as having lower standing. When *i* adopts the dominant strategy of donating to those with a similar or better reputation, then *j* must increase her own reputation (i.e., number of donations) in order to remain eligible to receive donations from *i*. We note that this dynamic operates within each generation, between selection and reproduction. Social comparison couples individual perception of others to their own standing, and evolution acts on the heuristics governing relative perceptions, rather than on absolute thresholds for the perception of acceptable/unacceptable donation behaviour.

**Figure 2:**
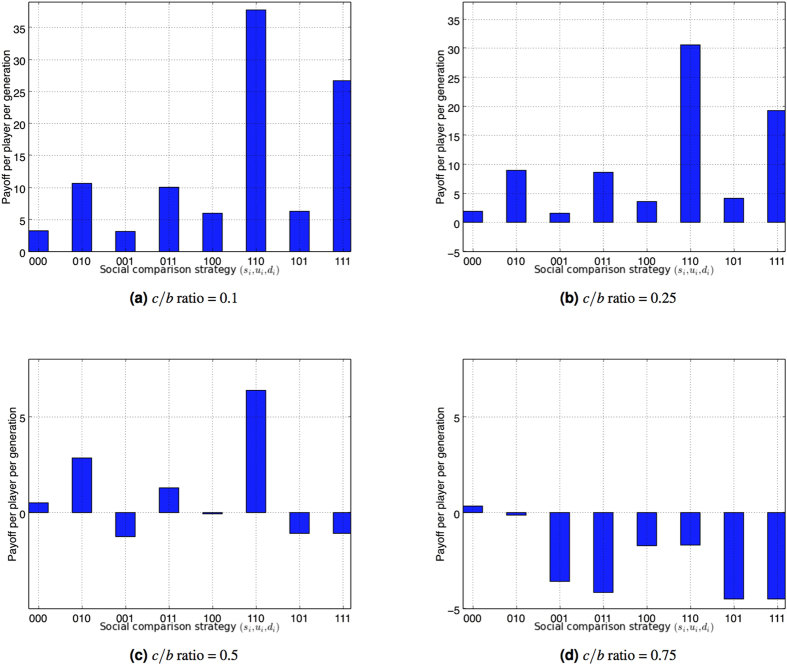
Average payoff per player per generation for the alternative social comparison strategies, using image scoring assessment while varying the cost-benefit ratio *c*/*b*. Parameter settings are consistent with those in Fig. 1.

Figure 2 shows the results from Fig. 1 in terms of average payoff per player per generation, where the payoff to an individual adopting a given strategy is the difference between benefit and cost incurred over a generation. For lower cost-benefit ratios (e.g., 0.1, 0.25) that support the emergence of cooperation, the payoff per individual reflects the behaviour in Fig. 1 where the cooperative strategies produce the highest payoff, and in particular the dominant approach of donating to those with similar or higher reputation. When the cost-benefit ratio reaches 0.5 this trend is reversed. The dominant (1, 1, 0) heuristic still produces the highest payoff per individual but with marginal average payoff as compared to lower *c*/*b* ratios. Beyond this *c*/*b* ratio (i.e., *c*/*b* = 0.75), defection becomes rational (Fig. 1c) but yields little positive payoff on average. Here the vast majority of generations are characterised by near zero donations being made.

### Discriminatory assessment rules reinforce the dominant strategy

**Figure 3:**
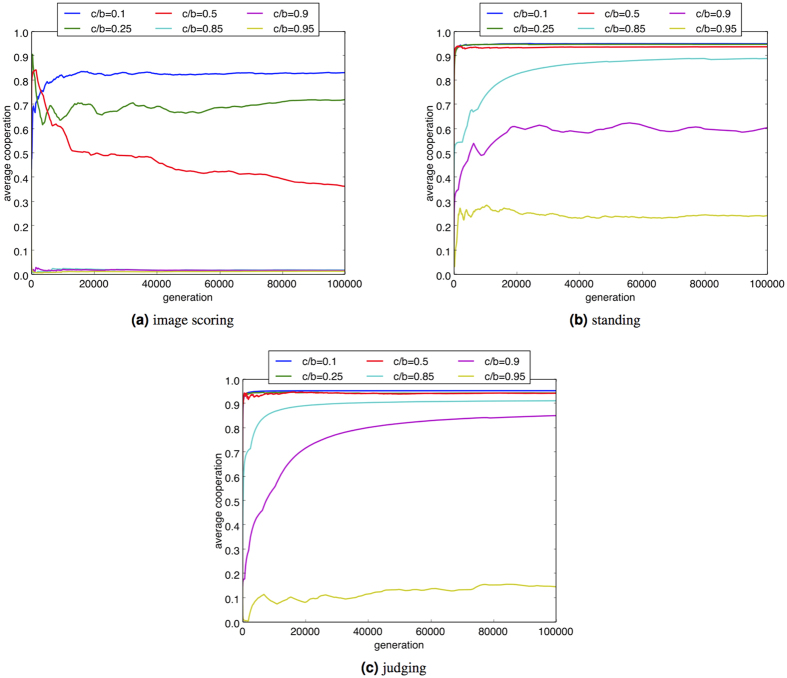
Cooperation from the social comparison strategies using different assessment rules while varying the cost-benefit ratio *c*/*b*. Parameter settings are consistent with Fig. 1. “Average cooperation” indicates the frequency of cooperative interaction: the number of donations made as a proportion of the total number of games played in all preceding generations.

The evolution of indirect reciprocity under image scoring is known to be susceptible to non-discriminatory assessment rules^3^,^57^ and therefore it is valuable to consider the effects of standing and judging^2^,^54^ to update reputation (Fig. 3). When generalised to a non-binary representation of reputation and considered in the context of social comparison, standing involves decrementing the reputation of *i* when *i* defects in light of a request from a player *j* with at least the reputation of *i*. Judging offers greater penalisation than standing by punishing a donor for not further targeting their behaviour, with the reputation of *r*_*i*_ decremented when *i* makes a donation to a less reputable recipient *j*.

We observe that the discrimination provided by standing and judging exactly represents penalties for actions which are inconsistent with the dominant social comparison rule of donation to a recipient of similar or upward standing. Consequently the social norms provided by standing and judging embody social comparison and this mechanism further supports the evolution of indirect reciprocity, as seen in Fig. 3. In particular standing and judging increase the extent of cooperative behaviour in the population, reaching in excess of 90% for low cost-benefit ratios (e.g., 0.1). The selective effects of discrimination from standing and judging, as compared to image scoring, also significantly extend the range of cost benefit ratio at which cooperation is sustained, for example with both standing and judging reaching nearly 90% cooperation levels with cost-benefit ratios of 0.85. Thus when the cost is relatively high, discrimination becomes influential.

### Social comparison provides robustness against errors

We investigate the sensitivity of the social comparison model to errors in both user perception and execution. Perception errors involve inaccuracy in the perceived reputation, modelled by misreading the potential recipient’s reputation with probability *p*_*r*_, in which case an alternative reputation is uniformly selected from another member of the population. This type of error has been a focus for attention in previous studies^2^, aligned to the effects of gossip and malicious misreporting^5^. Perception error is known to cause negative effects on discriminatory assessments such as standing^58^, but exhibiting robustness when error rates are relatively small^17^.

**Figure 4:**
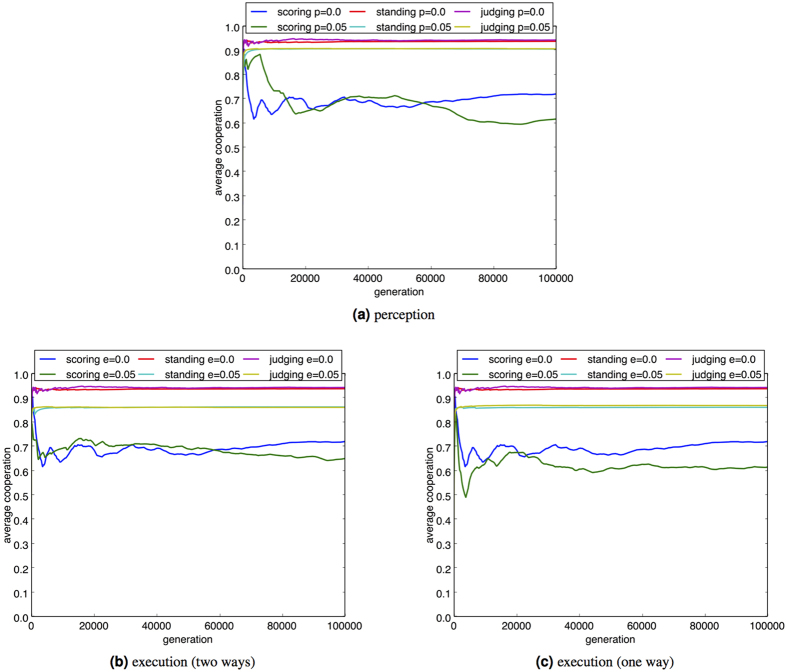
Effect of perception and execution error on the social comparison strategies. *c*/*b* ratio = 0.25. Parameter settings are consistent with Fig. 1. The error rate applied is 5%.

Results (Fig. 4) are consistent with previously published work applying perception error^17^. When applying standing and judging for social comparison, evolution is resilient to reasonable error rates such as 5% with similar degradation in the frequency of cooperative interaction evident when the experiment is repeated at a higher error rate (e.g., *p*_*r*_ = 10%). Image scoring exhibits similar behaviour under perception error but shows a large degradation in the population’s cooperative behaviour as error level increases.

In contrast to perception error, execution errors represent involuntary human mistakes, which have received less attention^3^,^59^. This error represents a failure to execute the intended strategy and has two forms: one-way execution error is applied with probability *e* to any donation action; two-way execution error is applied with probability *e* to both donation and defection decisions. Consistent with the published literature^17^, results from our experiments show that strategies based on social comparison are robust to modest errors of both types (e.g., *e* = 5%). However, the impact of execution errors on the frequency of donation is generally worse than perception errors, increasing with the error rate. Additionally, the discriminating strategies of standing and judging show almost identical characteristics for both one-way and two-way errors. With perception errors there is a chance that reputation will still be appropriately classified by social comparison, however failure to execute an intended action offers no direct opportunity for evolutionary recovery through rebalancing effects, that is errors leading to increased defection being offset by errors leading to increased cooperation.

For non-discriminating assessment provided by image scoring, the results from the two-way execution error not only exhibits superior cooperation levels as compared to one-way execution error, but the results are comparable to those of standing and judging in terms of the decrease in average cooperation as compared to a zero error state. This is consistent with the observation that two-way execution error may self-compensate through the equal treatment of error in defection and donation^3^,^59^ which is more likely to occur when reputation is updated without discrimination, as in image scoring.

### The dominant social comparison heuristic provides evolutionary stability

**Figure 5:**
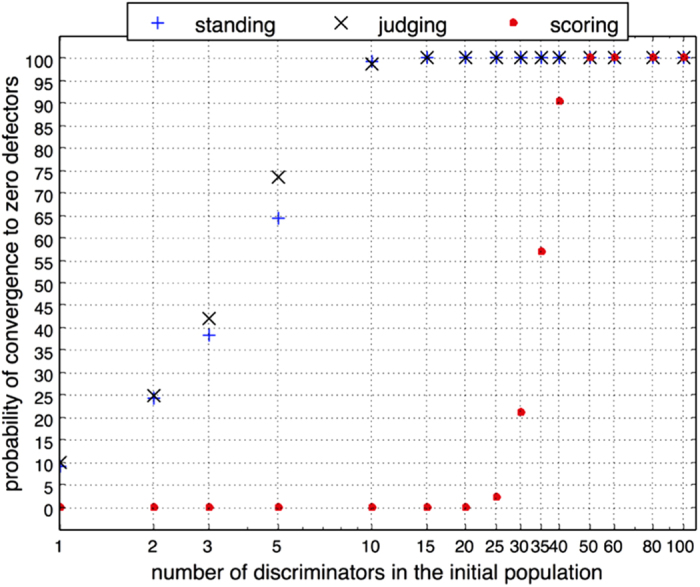
The ability of a discriminating sub-population adopting the (1, 1, 0) heuristic, to dominate in the presence of defectors. Population size *N* is fixed at 100. *c*/*b* ratio = 0.25. *μ* = 0. Other parameter settings are consistent with Fig. 1. Error rates in both execution and perception are applied at 5%. The probability of convergence to zero defectors represents the proportion of cases from 1000 runs in which the behaviour is observed.

We assess the dominant similar and upward comparison heuristic for evolutionary stability. As with all other strategies, strategies involving social comparison cannot discriminate against duplicitous agents who initially cooperate to encourage the evolution of a prosocial population, with a view to subsequently exploiting the population by free-riding^60^. However, it is prudent to examine the extent to which discrimination present in the similar and upward comparison heuristic is sufficient to dominate over defectors, including performance in extreme scenarios. This is shown in Fig. 5, where a sub-population adopting the similar and upward comparison heuristic is examined in the presence of defectors. The sub-population adopts this heuristic with assessment through either image scoring, standing or judging. The probability of convergence to zero defectors represents the proportion of cases from 1000 runs.

Overall, a high proportion of defectors are needed to prevent the evolution of a sub-population that adopts the dominant (1, 1, 0) heuristic. If just 10% of the population apply the similar and upward comparison heuristic while discriminating through standing or judging, then the chance of fully eradicating defectors is 98.7% for standing and 99.2% for judging. Consistent with previous observations made on the lesser evolutionary stability of image scoring^3^, a much larger sub-population is required (over 40%) to achieve similar levels of performance when image scoring is applied as the assessment rule. These results suggest considerable resilience, particularly when the social norm in standing and judging further reinforces behaviour consistent with similar and upward comparison.

Convergence to zero defectors is relatively rapid even when the initial sub-population adopting the similar and upward comparison heuristic is small. For example, on average, when adopting standing within a sub-population representing 5%, the population converges to zero-defectors within 10.55 generations (*SD* = 3.45), where each player acts as a potential donor on average 50 times per generation. Under the same conditions judging converges marginally quicker (mean = 10.3, *SD* = 3.37) and image scoring never converges to a population with zero defectors. With a sub-population of 40 adopting the similar and upward comparison heuristic, there is a greater chance that the population will converge to zero defectors. This occurs more slowly for image scoring (mean = 7.63, *SD* = 2.24) as compared to standing (mean = 3.61, *SD* = 0.69) and judging (mean = 3.52, *SD* = 0.69).

### A hetrogeneous population structure can enhance the global cooperation level

We assume a heterogeneous population structure by sub-dividing the population into isolated social groups consistent with the idealised Island Model^17^. The social groups define the boundaries within which members may donate to others. The global population (*N* = 100) is structured into *g* social groups of equal size for *g* = 2, 3, 4, 5 (when *g* = 3 the groups are of size 33 and 34). We adopt assessment by image scoring and standing with *c*/*b* ratios selected as 0.1 and 0.85 respectively, and execution and perception error rates of 2.5% are applied. These conditions allow the observation of a heterogeneous population when *p*, the probability of reproduction from the local sub-population rather than the global population, is varied.

**Figure 6:**
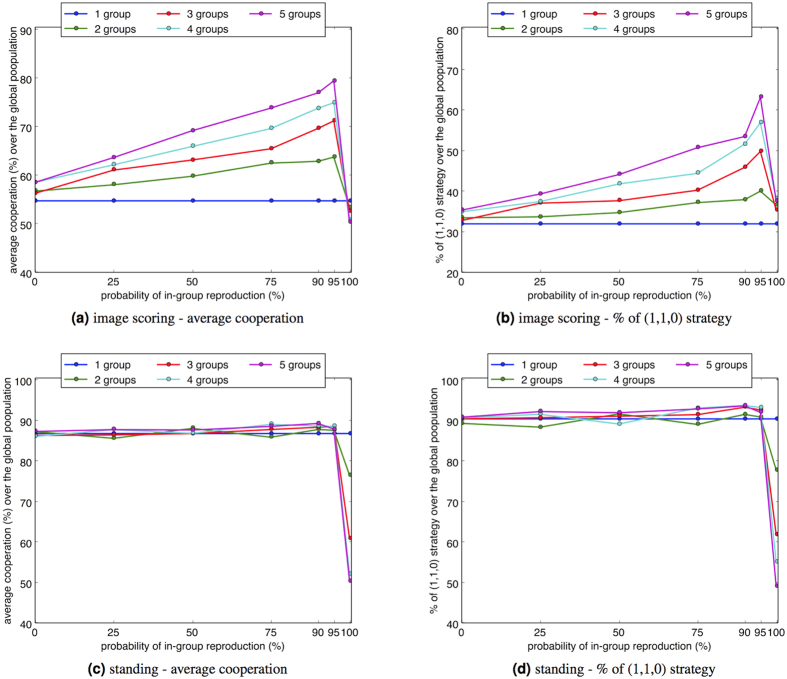
Average cooperation level and percentage of the (1, 1, 0) heuristic from all games in all generations, applying a heterogeneous population with *g* groups, for *g* = 1, 2, 3, 4, 5. *c*/*b* ratio for image scoring is 0.1. *c*/*b* ratio for standing is 0.85. Perception and execution errors are applied, both with a rate of 2.5%. Other parameter settings are consistent with Fig. 1. “Average cooperation” indicates the frequency of cooperative interaction: the number of donations made as a proportion of the total number of games played.

Under these parameters the results show that a social group structure can positively affect the evolution of cooperation. This is particularly evident for the less sophisticated image scoring assessment, as compared to standing, where potential increases in cooperation are at best marginal. Figure 6 shows that for image scoring cooperation increases with both the number of social groups and the probability of reproduction within groups *p*. However, when reproduction is entirely limited to the local population (*p* = 1), total cooperation levels drop significantly, with smaller groups increasing this effect for both image scoring and standing.

Contributory to this phenomenon is the small number of possible strategies that social comparison affords, with just eight possible states as compared to 121 for the original image scoring model^1^. This encourages dominant strategies to readily evolve in small sub-groups, although such dominant strategies may be non-cooperative due to the lower chance of in-group diversity and the effects of genetic drift. However when a small chance of reproduction from the global population is introduced (e.g., *p* = 0.95), this provides an opportunity to introduce, with high payoff, cooperative strategies into any non-cooperative sub-groups. As found in the previous section of results, only a small number of players with number of the (1, 1, 0) strategy are required to dominate over a defecting population, allowing non-cooperative sub-groups to be dominated. The results in Fig. 6 also reaffirm the correlation between the dominant (1, 1, 0) social comparison heuristic and high cooperation levels.

## Discussion

The results demonstrate that heuristics based on social comparison support the evolution of indirect reciprocity, naturally implying eight possible heuristic alternatives. Critically, each heuristic is based on relative evaluation to oneself, in alignment with evidence of a human psychological disposition. This means that an individual’s reputation may also affect their perception of others, in contrast to reputation systems that are often solely focussed on how they may be perceived by others.

The results show that a dominant social comparison heuristic is readily identifiable, namely donating to those that are at least as reputable as oneself. This is a form of *aspirational homophily*, since it represents association, through donation, with those of similar or preferential reputational status. Adopting a strategy incorporating this heuristic supports a phenomenon where to remain eligible for donations from reputable peers, recipients must also maintain their own reputation. Because social comparison heuristics assume that perceptions are made relative to oneself, this dynamic functions within each generation of evolution, meaning that an individual’s eligibility to receive or make a donation may change even though their strategy could remain fixed. Through these comparative interactions, an individual’s donation behaviour and prospects to receive a donation are influenced by others, being dependent on the reputation of the wider population.

We note that a number of experiments concerning human behaviour provide indirect empirical insights on the dynamics that we observe through simulation. Cooperation in the form of generosity has been observed to be contagious^61^, with receipt of donations positively influencing their subsequent generosity. Observational evidence^62^ suggests that the image score of the recipient influences the helping decision, with a reasonable number of participants identified as making this decision relative to their own image score. Homophilic donation behaviour has been observed^63^ where high donors achieve a higher than average expected payoff by cooperating mainly with other highly cooperative donors. Similar findings are also present in the context of combined global social and reputational knowledge^64^, where cooperators form a separate community that achieves a higher cooperation level than the community of defectors. These observations point to the behavioural relevance of comparison and reputational homophily in sustaining possible cooperation.

In common with other models, in addition to specifying heuristic conditions for donation, social comparison strategies must define assessment rules that provide criteria for updating reputation in response to donation. Applying standing or judging with social comparison heuristics has a significant positive effect on evolutionary stability, enabling small numbers of individuals to discriminate against defectors and dominate through successive reproduction. While the assessment rules of standing and judging have previously been observed as effective in reinforcing the evolution of indirect reciprocity, such as by providing additional discrimination over image scoring^2^,^3^, we observe that both standing and judging operate by penalising actions that are inconsistent with the dominant social comparison heuristic of donation to those whose reputation is similar or upward in comparison. This has not been previously observed, and is a possible contributory factor in making the discrimination of standing and judging effective, by ensuring that conditions are supported that don’t impede natural selection.

We also observe that the dominant social comparison heuristic is a pre-requisite for the evolution of indirect reciprocity identified in significant previous contributions. Nowak and colleagues^1^ showed that evolution based on image scoring could favour indirect reciprocity. The evolution of a pair of absolute reputation-based thresholds *h*, *k* were observed, where *i* donates to *j* if *j* has an image of at least *k* and/or *i*’s own image is less than *h*. Notably the dominant social comparison heuristic is immediately evident: threshold *k* supports donation by *i* when similar and upward comparison with the reputation of *j* is observed. Additionally the dominant social comparison heuristic is also implicitly present in the results: Fig. 4(a,b)^1^ show that strategies cannot significantly evolve when *h* < *k*, which is precisely the when overlap between the donor and recipient images is not possible. When this is relaxed, it then becomes possible for similar and upward comparison between the donor’s target image (i.e., threshold *h*) and the minimum threshold on donating to the recipient (i.e., *k*), representing the region where significant evolution is observed (Fig. 4(a,b)^1^).

These observations indicate that the dominant social comparison heuristic may play a more general role in supporting the evolution of indirect reciprocity. The most comprehensive understanding of the evolution of indirect reciprocity has been obtained when reputation is assumed to be binary. Binary reputation assumes simplified cognition, where members of a population view others as having either a ‘good’ or ‘bad’ standing, as originally modelled from an economic perspective^9^. Through this simplification, it has been possible to consider all options for assessment of reputation and donor action^52^. Exactly eight possibilities for evolutionary stable assessment have been identified^4^: thus under assumptions of a binary reputation, these results precisely capture the conditions where indirect reciprocation can be robustly sustained (Table 1).

Table 1 shows that when the donor *i* and the recipient *j* are both in bad standing (i.e., *i* = 0, *j* = 0), assessment rules and donation decisions are irrelevant, leaving three combinations of donor-recipient reputation (i.e., *i* ≠ 0 ∨ *j* ≠ 0). The view of the recipient’s reputation, from the donors perspective, can be interpreted in terms of social comparison (far right column, Table 1), and when doing so, we observe that the associated stable actions for donor *i* exactly correspond to the dominant social comparison heuristic: agent *j* donates when and only when recipient *i* has a similar or higher reputation. Thus, under binary reputation, the dominant social comparison heuristic exactly models the optimal actions.

In summary, simple self-referential cognitive approaches to decision making and the evolution of indirect reciprocity appear to be strongly linked. From a behavioural perspective, the origins of social comparison are potentially distant^40^, and belie survival related decision-making. Social comparison features as a way in which individuals comprehend and reason about their place within society^65^. Significant evidence indicates that while humans may lack the capacity to rationally evaluate the huge number of decisions that they face^27^, heuristics characterise the intuitive thinking that compensates^66^. Recent work^22^ has shown that intuitive decision making in cooperative one-shot dilemmas may generally be guided by social heuristics that reinforce previously successful behaviour, with slower reflexive processes moderating fitness of the heuristic to the wider context. Given that relative positioning within social context affects donation behaviour^35^,^36^,^37^, actions based on social comparison are immediate candidates for social heuristics.

Social comparison heuristics also provide an interesting perspective on conditions supporting the evolution of indirect reciprocity. Beyond recent contributions^22^,^26^, behavioural consideration of prosociality has largely occurred in isolation from the characterisation of such conditions. However through associated heuristics, social comparison naturally lends itself to evolutionary analysis, and the social comparison heuristic of donating to those with similar or a higher reputation dominates, which is consistent with social comparison being a form of evaluation for aspirational human behaviour^67^. Leading observations on the evolution of indirect reciprocity^1^,^4^,^9^ have connection to the dominant social comparison heuristic, to the extent that under binary representation this heuristic exactly characterises the actions of the evolutionary stable solutions. Furthermore, discriminatory social norms for crediting individuals with reputation, in particular standing and judging, represent penalisation for actions that are inconsistent with the dominant social comparison heuristic.

Given that social comparison heuristics provide insight into the explanation for conditions supporting indirect reciprocity, an extraordinary feature of humans in contrast to other species, we note that any social comparison involved could have also influenced the evolution of the social brain. As implied by the social brain hypothesis^41^,^68^,^69^, living in functional social groups imposes cognitive demands that are consistent with the evolution of species having a larger relative brain size^70^. These cognitive demands stem from the information processing associated with the social complexity of larger groups^71^. It has been conjectured^18^ that indirect reciprocity may have provided the selective challenge driving the cerebral expansion in human evolution, albeit without reference to a candidate mechanism. As social comparison is evident in the evolution of indirect reciprocity, that it is prevalent in observed human behaviour and that human survival through sociality is enhanced by indirect reciprocity, we conjecture that social comparison has provided sufficient difficulty to promote such cerebral expansion, consistent with the social complexity hypothesis^72^.

We also note that these findings also have wider relevance for contemporary autonomous systems^73^. Beyond human intelligence, the aspirational homophily heuristic has implications for the evolution of distributed computational and communication systems that involve one-shot interactions. Recent examples include device to device communication for opportunistic networks^74^,^75^, which can be supported by exploiting cooperative protocols between devices or their users^76^,^77^. We note that subject to an accurate means of third party perception, social comparison lends itself to machine execution, opening up prospects for autonomous entities to pursue optimal behaviour based on a simple heuristic of relative self comparison, which supports both individual and social utility with limited requirements for centralised control.

## Methods

We apply an evolutionary framework based on the donation game, a special case of the mutual aid game^59^ assuming a single donor. Parameter settings represent typical conditions through which the effects of social comparison are observable. Unless otherwise stated, results represent an average of 5 randomly seeded observations. Information on accessing data supporting the results is available^78^.

### Evolution

Unless otherwise stated in the experiment, we apply a single homogeneous population of *N*-players over *M*-generations, with *m* games per generation, and we use default parameters of *N* = 100, *M* = 100,000 and *m* = 5,000, resulting in each player participating in an average of 50 games per generation. A heterogeneous population is adopted using the modified Island Model^17^, where the global population of 100 is divided into *g* social groups of equal size (when *g* = 3 the groups are of size 33 and 34). In each game a donor *i* and potential recipient *j* are selected at random. When the population is heterogeneous, *j* is always randomly selected from the same group as *i*.

### Action rules

Evolution acts upon individual social comparison heuristics, which for a donor *i* is denoted by a binary triple (*s*_*i*_, *u*_*i*_, *d*_*i*_) indicating whether or not *i* donates when similarity (*s*_*i*_), upward comparison (*u*_*i*_) or downward comparison (*d*_*i*_) is observed by *i* in respect of the potential recipient *j*’s reputation (*r*_*j*_), as compared to *i*’s reputation (*r*_*i*_). These represent the action rules, where approximate similarity is identified when *r*_*j*_ − Δ ≤ *r*_*i*_ ≤ *r*_*j*_ + Δ, upward self-comparison occurs when *r*_*j*_ > *r*_*i*_ + Δ, and downward self-comparison occurs when *r*_*j*_ < *r*_*i*_ − Δ. We apply a default setting of Δ = 0 for the standing and judging assessments and Δ = 1 for image scoring. Unless otherwise stated in the experiment, the initial population is formed from randomly selected social comparison heuristics.

### Assessment

When players are assumed to have a non-binary reputation, this is incremented/decremented by integer units in the range ±5 as in refs 1,17, dependent on the assessment rule. Three assessment rules feature in this study: image scoring, standing and judging. In image scoring, a potential donor *i*’s reputation is incremented if a donation is made to *j*, and decremented otherwise. Standing is interpreted as decrementing the reputation of *i* when *i* defects in light of a request from a player *j* that is at least as reputable as *i*. Additionally judging represents decrementing the reputation of *r*_*i*_ when *i* makes a donation to a recipient *j* of lower reputation. Reputation is set to zero at the beginning of each generation, and assumed to be public, visible to all members of the population.

### Selection and reproduction

The act of donation from *i* to *j* results in an economic transaction, with cost *c* to player *i* and benefit *b* to player *j*. The payoff to player *i* over a generation is their total benefit arising from donations received less the total cost of the donations they made. At the start of each generation, individual payoff is set to zero and used as the fitness function. Social comparison heuristics are propagated to the next generation based on uniform random selection of heuristics from some group of players *S*, weighted by the fitness of the members of *S*. This clonal reproduction is dependent on a single parent and commonly used in previous studies on indirect reciprocity based on evolutionary simulation^1^,^17^,^57^,^58^. When the population is assumed to be heterogeneous, *S* is the parent’s social group with probability *p*, and *S* is the global population with probability 1 − *p*. When the population is assumed to be homogeneous, *S* represents the global population. When creating a new generation, mutation allows a random change of heuristic to take place with probability *μ* = 1/100.

## Additional Information

**How to cite this article**: Whitaker, R. M. *et al*. A Dominant Social Comparison Heuristic Unites Alternative Mechanisms for the Evolution of Indirect Reciprocity. *Sci. Rep.* **6**, 31459; doi: 10.1038/srep31459 (2016).

## Figures and Tables

**Table 1:** The leading eight stable strategies and social comparison.

assumed initial reputations		stable assessments - updated reputation for *i* in response to:		stable actions for donor *i*	donor *i*’s comparative view of recipient *j*’s reputation
*i*	*j*	defection against *j*	donation to *j*		
1	1	0	1	**donate**	**similar**
1	0	1	any	**defect**	**lower**
0	1	0	1	**donate**	**higher**
0	0	any	any	donate or defect	similar
